# Supplementary figures and images for: Abundance of Phasi-Charoen-like virus in Aedes aegypti mosquito populations in different states of India
Source: PLoS One. 2022 Dec 9;17(12):e0277276. doi: 10.1371/journal.pone.0277276 (PMC9733876; doi:10.1371/journal.pone.0277276)

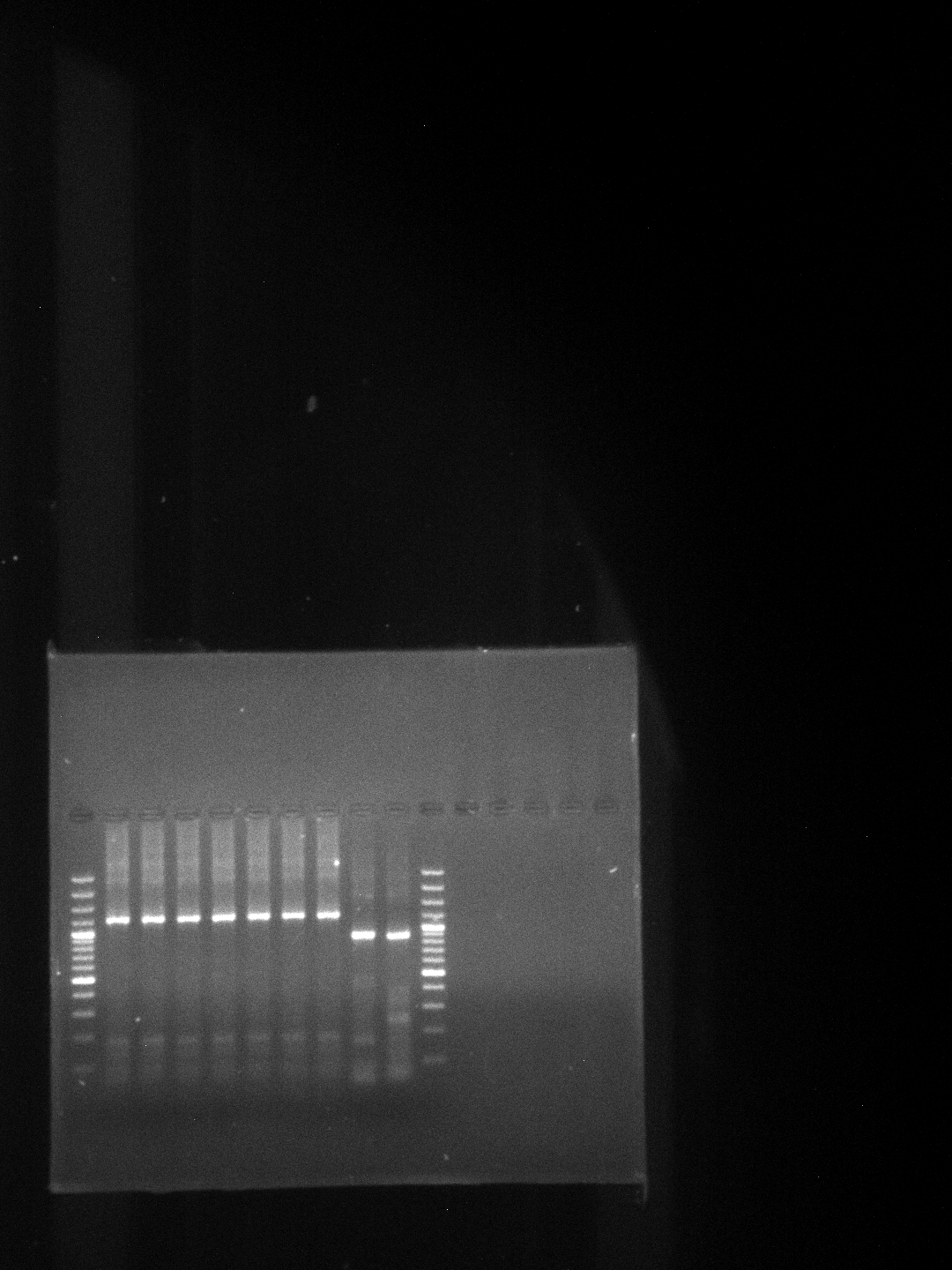

Supplement: S1 Fig — Products were analysed on 2% agarose gel, lanes- L1: Complete S segment, L2: Partial M segment, L3: Partial L segment, L4: 100 bp DNA ladder, L5: Negative control. (TIFF) [file pone.0277276.s001.tiff]

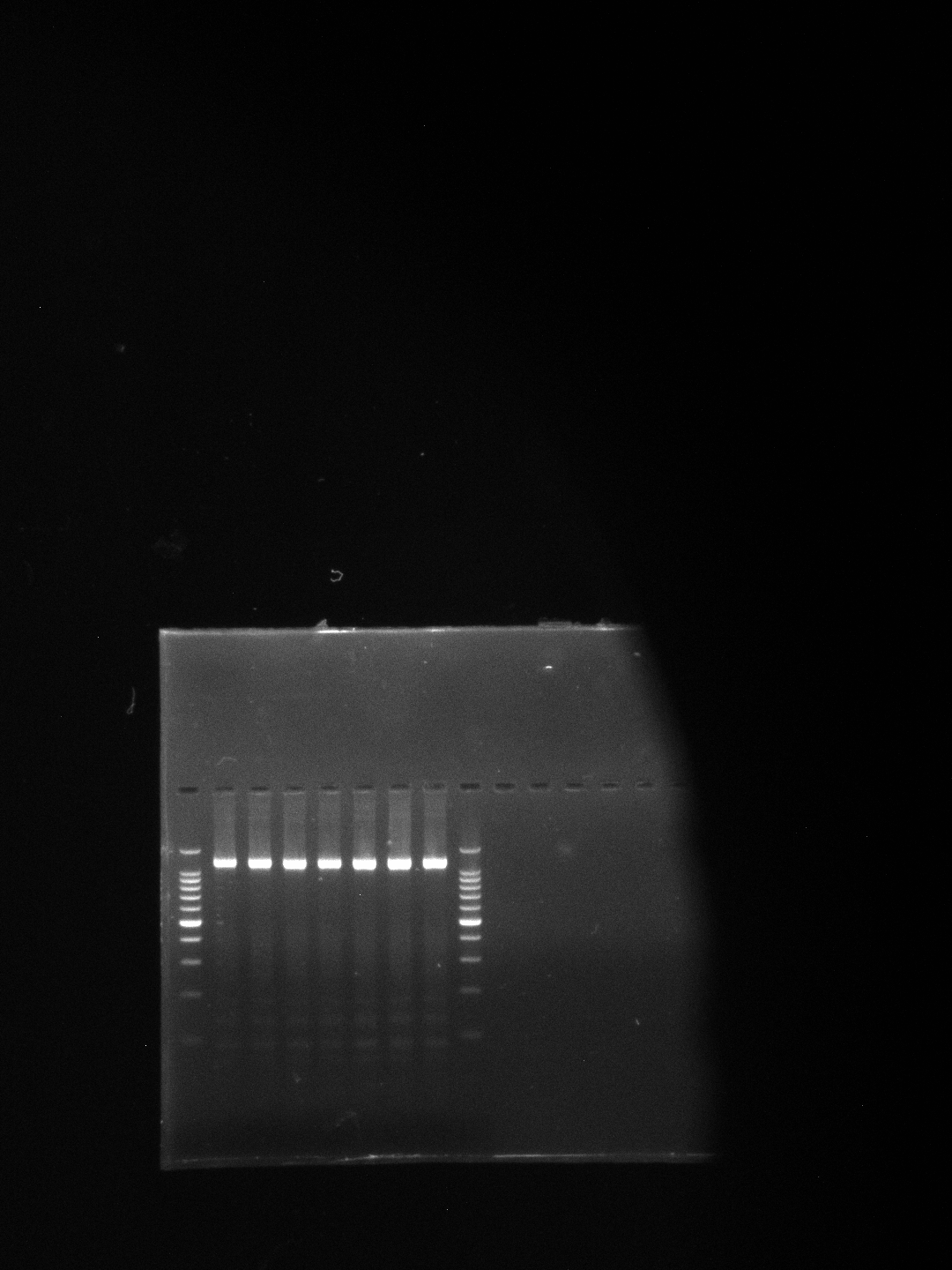

Supplement: S2 Fig — Products were analyzed on 2% agarose gel, lanes- L1: 100 bp DNA ladder (Invitrogen), L2: Assam (AS) adult mosquito pool, L3: Pune, Maharashtra (MH) adult mosquito pool, L4: Karnataka (KA) adult mosquito pool, L5: Tamil Nadu (TN) mosquito eggs, L6: Tamil Nadu (TN) mosquito larvae, L7: Tamil Nadu (TN) mosquito pupae, L8: Tamil Nadu (TN) mosquito adults, L9: 100 bp DNA ladder. (TIF) [file pone.0277276.s002.tif]
