# Supplementary material for: Abundance of Phasi-Charoen-like virus in Aedes aegypti mosquito populations in different states of India
Source: PLoS One. 2022 Dec 9;17(12):e0277276. doi: 10.1371/journal.pone.0277276 (PMC9733876; doi:10.1371/journal.pone.0277276)
Supplement: S1 Table — (DOCX) [file pone.0277276.s003.docx]

**Table S1: Reads of representative sequencing runs after basecalling, adaptor and primer trimming**

| Sample | Number of sequences | Total bases (Mb) | Minimum length | Average length | Maximum length |
| --- | --- | --- | --- | --- | --- |
| RUN2_barcode01 | 268056 | 92.73 | 100 | 346 | 2653 |
| RUN2_barcode03 | 222872 | 95.17 | 101 | 427 | 2475 |
| RUN2_barcode04 | 1018244 | 225.54 | 100 | 222 | 2146 |
| RUN2_barcode05 | 464826 | 125.58 | 100 | 270 | 2295 |
| RUN2_barcode06 | 851050 | 207.16 | 100 | 243 | 3151 |
| RUN2_barcode07 | 666057 | 159.19 | 100 | 239 | 2447 |
| RUN2_barcode08 | 595298 | 155.71 | 100 | 262 | 2821 |
| RUN3_barcode01 | 892 | 0.23 | 135 | 261 | 941 |
| RUN3_barcode02 | 2557 | 0.64 | 104 | 252 | 2708 |
| RUN3_barcode03 | 3525 | 0.88 | 101 | 249 | 1731 |
| RUN3_barcode04 | 4017 | 1.41 | 105 | 352 | 1486 |
| RUN3_barcode05 | 2047 | 0.73 | 100 | 355 | 1447 |
| RUN4_barcode01 | 1716557 | 705.58 | 100 | 411 | 2957 |
| RUN4_barcode02 | 1009577 | 313.58 | 100 | 310 | 2901 |
| RUN4_barcode03 | 744506 | 184.95 | 100 | 248 | 3753 |
| RUN4_barcode04 | 906849 | 333.91 | 100 | 368 | 3027 |
| RUN4_barcode05 | 1248237 | 351.51 | 100 | 281 | 3166 |
| RUN5_barcode01 | 615953 | 137.46 | 100 | 223 | 2211 |
| RUN5_barcode02 | 265149 | 68.54 | 100 | 258 | 1928 |
| RUN5_barcode03 | 319649 | 88.92 | 100 | 278 | 2673 |
| RUN5_barcode04 | 134302 | 32.51 | 100 | 242 | 1869 |
| RUN5_barcode05 | 3345 | 0.86 | 100 | 256 | 1557 |
| RUN5_barcode06 | 10087 | 6.92 | 100 | 686 | 3167 |
