# Supplementary material for: Abundance of Phasi-Charoen-like virus in Aedes aegypti mosquito populations in different states of India
Source: PLoS One. 2022 Dec 9;17(12):e0277276. doi: 10.1371/journal.pone.0277276 (PMC9733876; doi:10.1371/journal.pone.0277276)
Supplement: S2 Table — (DOCX) [file pone.0277276.s004.docx]

**Table S2: PCLV genome coverage in samples from different locations in Pune**

| **Sample** | ***Segment** | **Length of segment** | **Bases covered** | **Reads mapped** | **Coverage (%)** | **Mean depth** | **Mean base quality** | **Mean map quality** |
| --- | --- | --- | --- | --- | --- | --- | --- | --- |
| **RUN2**  **barcode01** | M | 3935 | 2993 | 1738 | 76.06 | 163 | 23 | 59.4 |
|  | L | 6806 | 5597 | 5192 | 82.24 | 253 | 23.5 | 59.1 |
|  | S | 1358 | 859 | 2699 | 63.25 | 744 | 23.1 | 59.8 |
|  | **Total** | **12099** | **9449** | **9629** | **78.10** |  |  |  |
| **RUN2**  **barcode03** | M | 3935 | 1644 | 321 | 41.78 | 45 | 22.7 | 60 |
|  | L | 6806 | 2144 | 775 | 31.50 | 33 | 23.6 | 58.5 |
|  | S | 1358 | 766 | 1709 | 56.41 | 403 | 22.9 | 59.7 |
|  | **Total** | **12099** | **4554** | **2805** | **37.64** |  |  |  |
| **RUN2**  **barcode04** | M | 3935 | 2805 | 425 | 71.28 | 25 | 23.3 | 58.5 |
|  | L | 6806 | 4798 | 150 | 70.50 | 6 | 23.5 | 54 |
|  | S | 1358 | 687 | 24 | 50.59 | 5 | 22.2 | 57 |
|  | **Total** | **12099** | **8290** | **599** | **68.52** |  |  |  |
| **RUN2**  **barcode05** | M | 3935 | 3407 | 857 | 86.58 | 59 | 23.3 | 58.2 |
|  | L | 6806 | 5292 | 1405 | 77.75 | 61 | 23.7 | 59 |
|  | S | 1358 | 700 | 620 | 51.55 | 135 | 23 | 59.6 |
|  | **Total** | **12099** | **9399** | **2882** | **77.68** |  |  |  |
| **RUN2**  **barcode06** | M | 3935 | 3224 | 2109 | 81.93 | 157 | 23.3 | 58.4 |
|  | L | 6806 | 4478 | 3169 | 65.79 | 130 | 23.5 | 58.8 |
|  | S | 1358 | 1211 | 3387 | 89.18 | 737 | 23 | 59.7 |
|  | **Total** | **12099** | **8913** | **8665** | **73.67** |  |  |  |
| **RUN2**  **barcode07** | M | 3935 | 3413 | 19459 | 86.73 | 1796 | 23.8 | 59.3 |
|  | L | 6806 | 5958 | 8872 | 87.54 | 627 | 23.2 | 58.2 |
|  | S | 1358 | 964 | 1454 | 70.99 | 536 | 24.3 | 59.2 |
|  | **Total** | **12099** | **10335** | **29785** | **85.42** |  |  |  |
| **RUN2**  **barcode08** | M | 3935 | 3028 | 9028 | 76.95 | 751 | 23.5 | 59 |
|  | L | 6806 | 5571 | 16374 | 81.85 | 884 | 23.5 | 58.9 |
|  | S | 1358 | 853 | 7756 | 62.81 | 2731 | 24.2 | 59.6 |
|  | **Total** | **12099** | **9452** | **33158** | **78.12** |  |  |  |
| **RUN3**  **barcode04** | M | 3935 | 2519 | 14 | 64.02 | 1 | 22.6 | 60 |
|  | L | 6806 | 3759 | 44 | 55.23 | 2 | 23.2 | 58.9 |
|  | S | 1358 | 654 | 43 | 48.16 | 11 | 23 | 59.1 |
|  | **Total** | **12099** | **6932** | **101** | **57.29** |  |  |  |
| **FIELD_RUN3**  **barcode05** | M | 3935 | 1397 | 6 | 35.50 | 1 | 22.7 | 53.2 |
|  | L | 6806 | 3334 | 20 | 48.99 | 1 | 24 | 60 |
|  | S | 1358 | 571 | 17 | 42.05 | 4 | 22.9 | 58.9 |
|  | **Total** | **12099** | **5302** | **43** | **43.82** |  |  |  |
| **RUN4**  **barcode01** | M | 3935 | 3752 | 7847 | 95.35 | 752 | 22.3 | 58.8 |
|  | L | 6806 | 6566 | 17707 | 96.47 | 996 | 22.7 | 58.9 |
|  | S | 1358 | 978 | 16449 | 72.02 | 4372 | 22.2 | 59.9 |
|  | **Total** | **12099** | **11296** | **42003** | **93.36** |  |  |  |
| **RUN4**  **barcode02** | M | 3935 | 3598 | 16094 | 91.44 | 1333 | 22.4 | 57.5 |
|  | L | 6806 | 4868 | 4903 | 71.53 | 233 | 22.6 | 57.4 |
|  | S | 1358 | 753 | 237 | 55.45 | 39 | 23.8 | 53.3 |
|  | **Total** | **12099** | **9219** | **21234** | **76.20** |  |  |  |
| **RUN4**  **barcode03** | M | 3935 | 3753 | 1192 | 95.37 | 94 | 22.3 | 57.3 |
|  | L | 6806 | 5897 | 658 | 86.64 | 34 | 22.5 | 57.8 |
|  | S | 1358 | 1070 | 60 | 78.79 | 9 | 23.7 | 55.2 |
|  | **Total** | **12099** | **10720** | **1910** | **88.60** |  |  |  |
| **RUN4**  **barcode04** | M | 3935 | 3447 | 6012 | 87.60 | 451 | 22.8 | 58.4 |
|  | L | 6806 | 3468 | 1047 | 50.96 | 47 | 22.8 | 57.8 |
|  | S | 1358 | 905 | 253 | 66.64 | 41 | 22.5 | 56.8 |
|  | **Total** | **12099** | **7820** | **7312** | **64.63** |  |  |  |
| **RUN4**  **barcode05** | M | 3935 | 3339 | 1238 | 84.85 | 82 | 22.5 | 56 |
|  | L | 6806 | 4320 | 1167 | 63.47 | 44 | 22.9 | 58.4 |
|  | S | 1358 | 750 | 476 | 55.23 | 105 | 23.8 | 59.4 |
|  | **Total** | **12099** | **8409** | **2881** | **69.50** |  |  |  |
| **RUN5**  **barcode01** | M | 3935 | 3766 | 438 | 95.71 | 36 | 23.5 | 58.3 |
|  | L | 6806 | 4377 | 269 | 64.31 | 14 | 24 | 58.4 |
|  | S | 1358 | 487 | 6 | 35.86 | 1 | 25.8 | 59 |
|  | **Total** | **12099** | **8630** | **713** | **71.33** |  |  |  |
| **RUN5**  **barcode02** | M | 3935 | 3521 | 507 | 89.48 | 43 | 23.9 | 58.6 |
|  | L | 6806 | 4879 | 503 | 71.69 | 27 | 23.9 | 57 |
|  | S | 1358 | 1166 | 40 | 85.86 | 11 | 23.7 | 57.9 |
|  | **Total** | **12099** | **9566** | **1050** | **79.06** |  |  |  |
| **RUN5**  **barcode03** | M | 3935 | 3740 | 527 | 95.04 | 46 | 23.3 | 58.5 |
|  | L | 6806 | 6340 | 1237 | 93.15 | 58 | 23.8 | 58.5 |
|  | S | 1358 | 1265 | 574 | 93.15 | 150 | 23.5 | 59.9 |
|  | **Total** | **12099** | **11345** | **2338** | **93.77** |  |  |  |

*****Reference sequences used for mapping reads is PCLV isolate Rio**,** segment M glycoprotein gene: NC_038261.1 (complete cds), segment L RNA-dependent RNA polymerase gene: NC_038262.1 (complete cds), segment S Nucleocapsid gene: NC_038263.1 – (complete cds)
