# Supplementary material for: Abundance of Phasi-Charoen-like virus in Aedes aegypti mosquito populations in different states of India
Source: PLoS One. 2022 Dec 9;17(12):e0277276. doi: 10.1371/journal.pone.0277276 (PMC9733876; doi:10.1371/journal.pone.0277276)
Supplement: S3 Table — (DOCX) [file pone.0277276.s005.docx]

**Table S3: GenBank accession numbers for ‘S’ segment sequences and sample details**

| Place of origin | Life cycle stage of | GenBank accession No. |
| --- | --- | --- |
| Tamil Nadu | Adult *Ae. aegypti* female | ON221813 IND (TN) |
| Tamil Nadu | *Ae. aegypti* larve | SRR18459033 - IND (TN) |
| Tamil Nadu | *Ae. aegypti eggs* | ON394047 - IND (TN) |
| Tamil Nadu | *Ae. aegypti pupae* | ON394046 – IND (TN) |
| Maharashtra | *Ae. aegypti* adult female | SRR18459031 - IND (MH) |
| Karnataka | *Ae. aegypti* adult male | SRR18459032 - IND (KN) |
| Assam | *Ae. aegypti* adult female | SRR18459030 - IND (AS) |
