# Supplementary material for: Abundance of Phasi-Charoen-like virus in Aedes aegypti mosquito populations in different states of India
Source: PLoS One. 2022 Dec 9;17(12):e0277276. doi: 10.1371/journal.pone.0277276 (PMC9733876; doi:10.1371/journal.pone.0277276)
Supplement: S1 File — (PDF) [file pone.0277276.s006.pdf]

Request for Permission to Publish Content under CC-BY License

Dear Rights Holder or Representative,

I have submitted a paper for publication in a PLOS journal, and wish to include the content listed below in the paper. I'm hereby requesting your (or your company's or institution's) permission to include the content in my paper. Please note that all PLOS journals are published under a Creative Commons Attribution License (CC BY), which allows for unrestricted use and distribution, even commercial, as long as attribution is given to the creator or rights holder of the content. See https://creativecommons.org/licenses/by/4.0/.

To grant me permission to use the content in my PLOS paper, please fill in the information below and then scan the completed form and send it to me at my email address.

Thank you.

My name:

Dr. Amol Nath, PhD student

My email address:

anniv271@gmail.com

Description of the content which I'm seeking permission to use (citation and/or title, and pasted screen shot, if applicable):

Map of Pune city in State- Maharashtra, Country- India

Link to the Content:

https://www.mapz.com/export/586388?view=download&with_layers=true?
next=/export/create?with_layers=true

* * *

On behalf of myself or the rights holder, I hereby grant the permission sought herein.

Signature of Party Granting Permission:

3470 25CC 852F 34BF 3C68 0B37 C523 6743 64DD 98EE

Date:

27. October 2022

Printed Name and Title:

Mr. Tim Kober, CEO

Kober-Kümmerly+Frey
Rolandstr. 83 · 50677 Köln · 0221/800334-0
info@mapz.com · www.mapz.com
